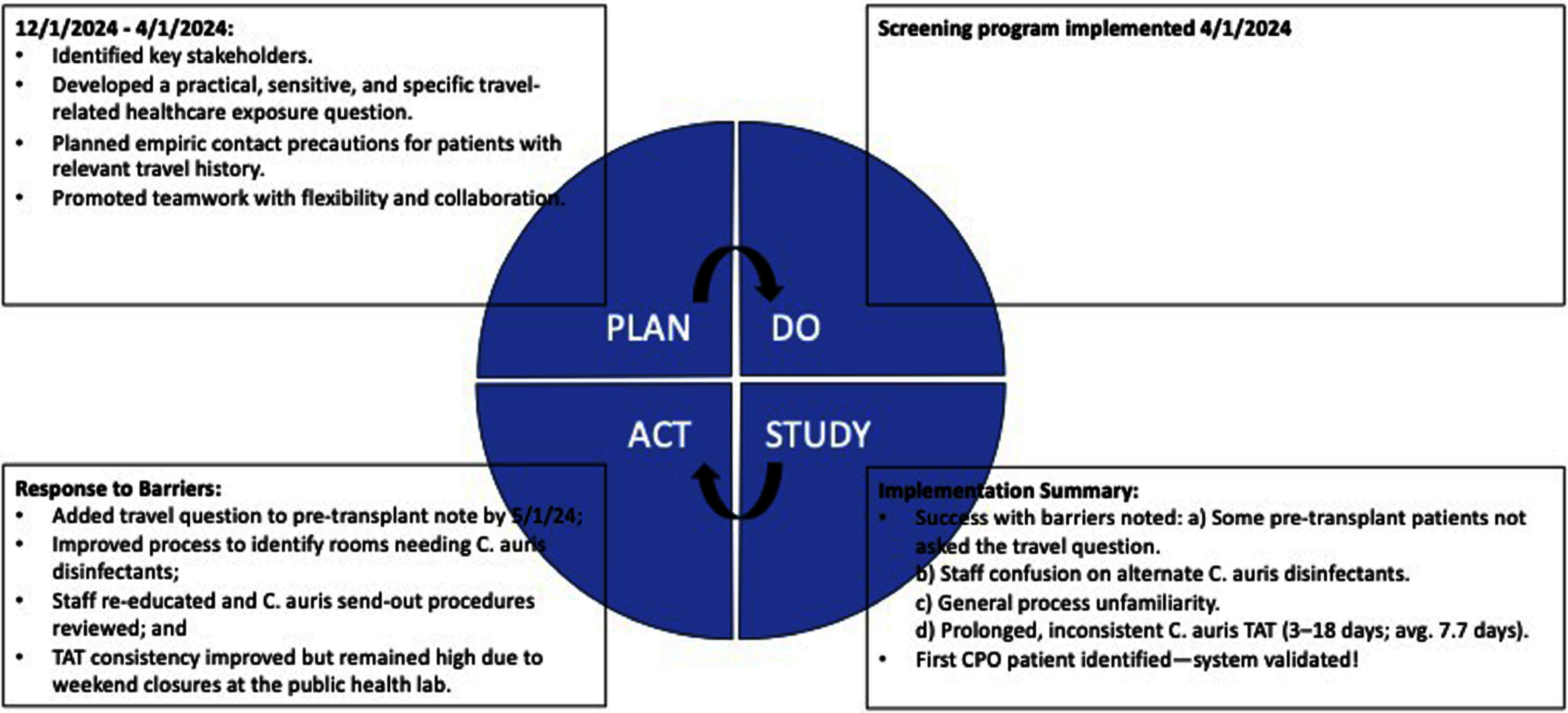# Candida auris and Carbapenemase-Producing Organisms Travel Screening: Program Implementation and Initial Report

**DOI:** 10.1017/ash.2025.394

**Published:** 2025-09-24

**Authors:** Katelyn West, Christopher Pfeiffer, Nicole Russo

**Affiliations:** 1 VA Portland Health Care System; 2 Portland VA Healthcare System

## Abstract

**Background:** Candida auris (C. auris) and carbapenemase-producing organisms (CPOs) are rapidly emergent healthcare-associated infections (HAIs) with high mortality. Early identification and isolation of colonized patients are crucial in preventing spread. Currently in Oregon, both organism types are uncommonly encountered such that local public health guidance advises travel-related screening as an important component of regional prevention. In 2024, VA Portland Health Care System (VAPORHCS) implemented a C. auris/CPO travel screening program as a quality improvement project. **Methods:** Using the Plan-Do-Study-Act (PDSA) framework, starting 4/1/2024 patients admitted to acute care were asked, “Have you had an overnight stay in a hospital, nursing home, or other healthcare facility outside of Oregon or Washington in the last year?” If patients responded affirmatively, the admitting nurse educated the patient and collected swabs after verbal consent: axilla/groin swabs for C. auris and peri-rectal swabs for CPOs. Patients were placed on empiric contact precautions in a single-bed room while awaiting results. Infection prevention prospectively monitored the implementation, and retrospectively medical records were reviewed. **Results:** The PDSA framework informed the implementation and helped organize the approach to addressing barriers such as missed screenings, communication breakdowns, complex disinfection protocols, the need for staff re-education, and delayed C. auris results (see Figure).

Of 3199 acute care admissions between 4/1/24–11/30/24, 72 patients (2.3%) reported a qualifying travel-related risk factor. 64 patients reported overnight healthcare elsewhere in the United States and Territories (including 5 in Puerto Rico) whilst 8 patients had international exposure (Mexico n=6, Philippines n=2). Of the 72 patients with qualifying travel, 9 patients were not tested (patient refused n=2, staff deemed inappropriate n=3, readmission n=1, unknown/technical issues n=3). An additional 32 patients (1%) initially reported qualifying travel but on chart review, travel was not confirmed. Of those, 16 had testing performed, all of which were negative. The average C. auris test turnaround time was 7.7 days with a range from 3-18 days. One patient (2.4%) tested positive for CPO. **Conclusion:** The C. auris/CPO screening program was effectively implemented and identified one positive CPO case, preventing the need for an urgent outbreak investigation. The PDSA framework helped the organization methodically plan the implementation and address barriers. The long turn-around-time for C. auris testing resulted in undesirable duration of empiric contact precautions. Continued evaluation of program metrics and public health recommendations are critical to sustainment and refinement over time.

**Figure:**